# 2,2-Bis(pyridin-2-yl)-1,3-diazinane

**DOI:** 10.1107/S1600536813007459

**Published:** 2013-03-23

**Authors:** Salim F. Haddad, Ismail Warad, Shehdeh Jodeh, Taibi Ben Hadda

**Affiliations:** a Department of Chemistry, The University of Jordan, Amman 11942, Jordan; b Department of Chemistry, An-Najah National University, Nablus, Palestinian Territories; c Laboratoire LCM, Faculté Sciences, Université Mohammed Ier, Oujda 60000, Morocco

## Abstract

In the title compound, C_14_H_16_N_4_, the six-membered hexa­hydro­pyrimidine ring adopts a chair conformation. In the crystal, one of the two pyrimidine N atoms engages in N—H⋯N hydrogen bonding with one of the pyridine N atoms, generating a helical chain running along the *c* axis. The helical pitch is the length of the *c* axis.

## Related literature

For related structures, see: Song *et al.* (2010 ▶); Jayaratna & Norman (2010 ▶); Fun & Kia (2008 ▶); Warad *et al.* (2012 ▶). For competition between cyclization and bis­imine formation, see: Locke *et al.* (2009 ▶). For the use of hexa­hydro­pyrimidines as polydentate ligands for the synthesis of transition metal coordination complexes, see: Schmidt *et al.* (2011 ▶). 
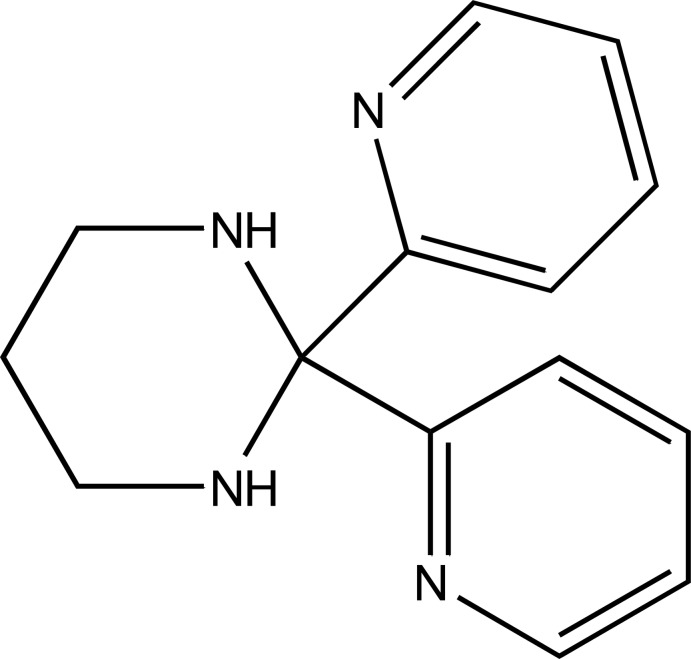


## Experimental

### 

#### Crystal data

C_14_H_16_N_4_
*M* *_r_* = 240.31
Monoclinic, 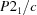

*a* = 14.2372 (14) Å
*b* = 8.0302 (8) Å
*c* = 11.3277 (9) Å
β = 103.075 (8)°
*V* = 1261.5 (2) Å^3^
*Z* = 4
Mo *K*α radiation
μ = 0.08 mm^−1^
*T* = 293 K
0.3 × 0.2 × 0.15 mm

#### Data collection

Agilent Xcalibur Eos diffractometer
Absorption correction: multi-scan (*CrysAlis PRO*; Agilent, 2011 ▶) *T*_min_ = 0.98, *T*_max_ = 0.99
4640 measured reflections
2238 independent reflections
1656 reflections with *I* > 2σ(*I*)
*R*_int_ = 0.023

#### Refinement

*R*[*F* ^2^ > 2σ(*F* ^2^)] = 0.041
*wR*(*F* ^2^) = 0.108
*S* = 1.05
2238 reflections
171 parameters
H atoms treated by a mixture of independent and constrained refinement
Δρ_max_ = 0.14 e Å^−3^
Δρ_min_ = −0.15 e Å^−3^

### 

Data collection: *CrysAlis PRO* (Agilent, 2011 ▶); cell refinement: *CrysAlis PRO*; data reduction: *CrysAlis PRO*; program(s) used to solve structure: *SHELXS97* (Sheldrick, 2008 ▶); program(s) used to refine structure: *SHELXL97* (Sheldrick, 2008 ▶); molecular graphics: *SHELXTL* (Sheldrick, 2008 ▶) and *ORTEP* (Burnett & Johnson, 1996 ▶); software used to prepare material for publication: *SHELXTL*.

## Supplementary Material

Crystal structure: contains datablock(s) I, global. DOI: 10.1107/S1600536813007459/nk2200sup1.cif

Structure factors: contains datablock(s) I. DOI: 10.1107/S1600536813007459/nk2200Isup2.hkl

Supplementary material file. DOI: 10.1107/S1600536813007459/nk2200Isup3.cml

Additional supplementary materials:  crystallographic information; 3D view; checkCIF report

## Figures and Tables

**Table 1:** Hydrogen-bond geometry (Å, °)

*D*—H⋯*A*	*D*—H	H⋯*A*	*D*⋯*A*	*D*—H⋯*A*
N3—H3⋯N2^i^	0.878 (17)	2.425 (18)	3.2845 (19)	166.4 (15)

Symmetry code: (i) 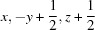.

**Fig. 1.**
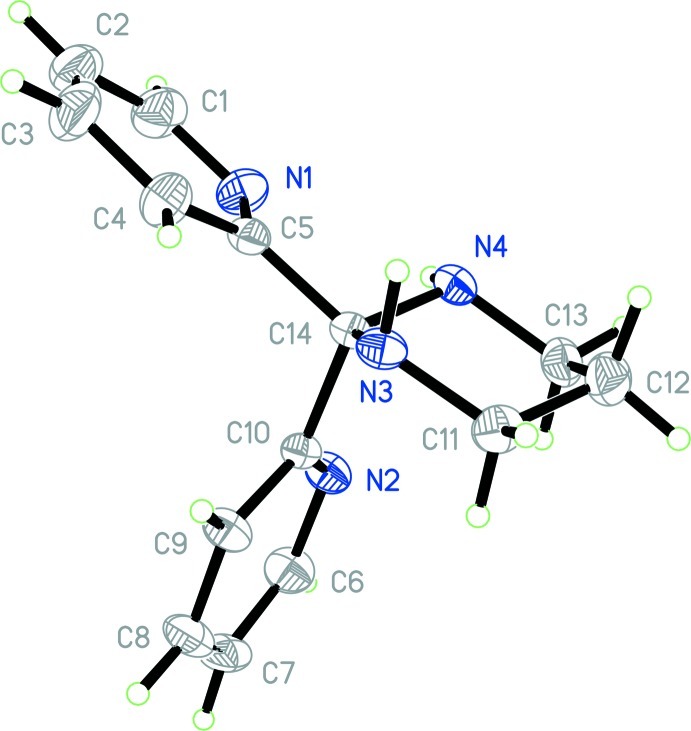
An ORTEP (Burnett & Johnson, 1996) view of the title compound. Displacement ellipsoids are drawn at the 30% probability level. H atoms are represented as small spheres of arbitrary radii.

**Fig. 2.**
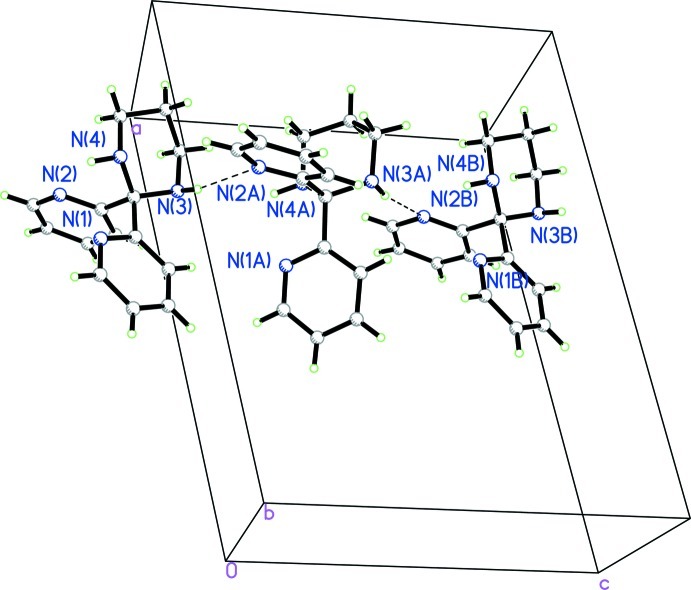
View approximately down b axis showing helical chains extending along c axis formed by intermolecular N—H···N hydrogen bonds joining one pyrimidinic nitrogen, N3, and one pyridinic nitrogen, N2 in adjacent molecule.

## supplementary crystallographic information

### Comment

Condensation of 1,3-diamines with 2-dipyridlketone is a well documented reaction for two potential products, hexahydropyrimidine and classical Schiff bases compounds (Warad *et al.*, 2012; Song *et al.*, 2010; Jayaratna & Norman, 2010; Fun & Kia, 2008). Both of these products are extensively utilized as polydentate ligands to synthesize coordination metal complexes. The title compound was obtained during our attempt to synthesize novel ligands in order to study their coordination chemistry.

The pyrimidine ring in the molecule assumes a chair configuration allowing better disposition for H-bonding. The molecular units are connected *via* hydrogen bonding between one pyrimidine nitrogen atom, N3 and one pyridinic nitrogen atom, N2 in adjacent molecule as shown in Fig. 2. Hydrogen bond values are tabulated.

### Experimental

A solution of 2-dipyridlketone (0.25 g, 1.45 mmol) in anhydrous ethanol (20 ml) was mixed with 1,3-propanediamine (0.16 ml, 1.5 mmoL) and allowed to reflux for about four hours. The resulting mixture was concentrated under reduced pressure and the title compound was precipitated by the addition of 40 ml of ice cool distilled water. The precipitate was filtered off, washed three times with 40 ml of distilled water, recrystallized in ethanol and allowed to stand at room temperature. After three days, colourless crystals suitable for single-crystal X-ray data collection were obtained (0.24 g, yield 77%).

### Refinement

All nonhydrogen atoms were refined anisotropically. H atoms attached to C were positioned geometrically, with C—H = 0.93 and 0.96 Å for aromatic and methyl H, respectively, and constrained to ride on their parent atoms, with *U*_iso_(H) = 1.2U_eq_. The two H atoms attched to the pyrimidinic N atoms were located in a difference Fourier map and refined fully to values of 0.88 (2) Å for the H atom involved in intermolecular H-bonding, and 0.92 (2) Å for the H atom involved in the biforcatd intramolecular hydrogen bonding. Highest difference peak and hole are 0.14 and -0.15 e/Å^3^ .

### Crystal data

**Table:**

C_14_H_16_N_4_	*F*(000) = 512
*M**_r_* = 240.31	*D*_x_ = 1.265 Mg m^−^^3^
Monoclinic, *P*2_1_/*c*	Mo *K*α radiation, λ = 0.71073 Å
Hall symbol: -P 2ybc	Cell parameters from 1403 reflections
*a* = 14.2372 (14) Å	θ = 3.1–29.1°
*b* = 8.0302 (8) Å	µ = 0.08 mm^−^^1^
*c* = 11.3277 (9) Å	*T* = 293 K
β = 103.075 (8)°	Parallelpiped, colourless
*V* = 1261.5 (2) Å^3^	0.3 × 0.2 × 0.15 mm
*Z* = 4	

### Data collection

**Table:**

Agilent Xcalibur Eos diffractometer	2238 independent reflections
Radiation source: Enhance (Mo) X-ray Source	1656 reflections with *I* > 2σ(*I*)
Graphite monochromator	*R*_int_ = 0.023
Detector resolution: 16.0534 pixels mm^-1^	θ_max_ = 25.0°, θ_min_ = 3.1°
ω scans	*h* = −16→15
Absorption correction: multi-scan (*CrysAlis PRO*; Agilent, 2011)	*k* = −9→9
*T*_min_ = 0.98, *T*_max_ = 0.99	*l* = −13→13
4640 measured reflections	

### Refinement

**Table:**

Refinement on *F*^2^	Primary atom site location: structure-invariant direct methods
Least-squares matrix: full	Secondary atom site location: difference Fourier map
*R*[*F*^2^ > 2σ(*F*^2^)] = 0.041	Hydrogen site location: inferred from neighbouring sites
*wR*(*F*^2^) = 0.108	H atoms treated by a mixture of independent and constrained refinement
*S* = 1.05	*w* = 1/[σ^2^(*F*_o_^2^) + (0.0446*P*)^2^ + 0.061*P*] where *P* = (*F*_o_^2^ + 2*F*_c_^2^)/3
2238 reflections	(Δ/σ)_max_ < 0.001
171 parameters	Δρ_max_ = 0.14 e Å^−^^3^
0 restraints	Δρ_min_ = −0.15 e Å^−^^3^

### Special details

**Table:**

Geometry. All e.s.d.'s (except the e.s.d. in the dihedral angle between two l.s. planes) are estimated using the full covariance matrix. The cell e.s.d.'s are taken into account individually in the estimation of e.s.d.'s in distances, angles and torsion angles; correlations between e.s.d.'s in cell parameters are only used when they are defined by crystal symmetry. An approximate (isotropic) treatment of cell e.s.d.'s is used for estimating e.s.d.'s involving l.s. planes.
Refinement. Refinement of *F*^2^ against ALL reflections. The weighted *R*-factor *wR* and goodness of fit *S* are based on *F*^2^, conventional *R*-factors *R* are based on *F*, with *F* set to zero for negative *F*^2^. The threshold expression of *F*^2^ > σ(*F*^2^) is used only for calculating *R*-factors(gt) *etc*. and is not relevant to the choice of reflections for refinement. *R*-factors based on *F*^2^ are statistically about twice as large as those based on *F*, and *R*- factors based on ALL data will be even larger.

### Fractional atomic coordinates and isotropic or equivalent isotropic displacement parameters (Å^2^)

**Table:**

	*x*	*y*	*z*	*U*_iso_*/*U*_eq_	
N3	0.79810 (10)	0.13990 (18)	0.07018 (11)	0.0369 (4)	
N2	0.79269 (10)	0.05042 (18)	−0.24206 (10)	0.0406 (4)	
N4	0.82606 (10)	0.32537 (17)	−0.08565 (12)	0.0377 (4)	
C14	0.76709 (11)	0.18825 (19)	−0.05728 (12)	0.0315 (4)	
C10	0.76853 (11)	0.03058 (19)	−0.13524 (12)	0.0306 (4)	
C5	0.66284 (12)	0.2490 (2)	−0.08265 (13)	0.0356 (4)	
C11	0.90172 (13)	0.1041 (2)	0.10491 (14)	0.0456 (5)	
H11A	0.9148	0.0036	0.0640	0.055*	
H11B	0.9198	0.0835	0.1915	0.055*	
C9	0.74371 (12)	−0.1230 (2)	−0.09704 (14)	0.0412 (4)	
H9A	0.7238	−0.1323	−0.0246	0.049*	
N1	0.63469 (11)	0.3400 (2)	−0.18294 (12)	0.0511 (4)	
C6	0.79561 (13)	−0.0872 (2)	−0.30852 (15)	0.0506 (5)	
H6A	0.8117	−0.0751	−0.3832	0.061*	
C13	0.92964 (13)	0.2875 (2)	−0.05846 (14)	0.0474 (5)	
H13A	0.9651	0.3833	−0.0774	0.057*	
H13B	0.9417	0.1947	−0.1078	0.057*	
C12	0.96337 (13)	0.2441 (3)	0.07420 (15)	0.0532 (5)	
H12A	0.9582	0.3410	0.1235	0.064*	
H12B	1.0304	0.2098	0.0911	0.064*	
C1	0.54414 (16)	0.3966 (3)	−0.20904 (18)	0.0639 (6)	
H1A	0.5240	0.4598	−0.2790	0.077*	
C7	0.77636 (14)	−0.2439 (2)	−0.27331 (15)	0.0520 (5)	
H7A	0.7821	−0.3359	−0.3210	0.062*	
C4	0.60138 (14)	0.2111 (3)	−0.00797 (16)	0.0539 (5)	
H4A	0.6222	0.1458	0.0608	0.065*	
C8	0.74829 (13)	−0.2622 (2)	−0.16589 (15)	0.0503 (5)	
H8A	0.7327	−0.3666	−0.1403	0.060*	
C2	0.47951 (15)	0.3674 (3)	−0.13943 (19)	0.0647 (6)	
H2A	0.4174	0.4109	−0.1607	0.078*	
C3	0.50821 (15)	0.2726 (3)	−0.03762 (19)	0.0685 (6)	
H3A	0.4655	0.2496	0.0114	0.082*	
H4	0.8042 (12)	0.352 (2)	−0.1662 (16)	0.052 (5)*	
H3	0.7859 (12)	0.224 (2)	0.1143 (14)	0.050 (5)*	

### Atomic displacement parameters (Å^2^)

**Table:**

	*U*^11^	*U*^22^	*U*^33^	*U*^12^	*U*^13^	*U*^23^
N3	0.0487 (9)	0.0332 (8)	0.0296 (7)	0.0036 (7)	0.0108 (6)	−0.0032 (6)
N2	0.0533 (10)	0.0376 (9)	0.0329 (7)	−0.0027 (7)	0.0141 (6)	−0.0042 (6)
N4	0.0455 (9)	0.0288 (8)	0.0398 (8)	−0.0039 (7)	0.0120 (6)	−0.0006 (6)
C14	0.0390 (10)	0.0265 (9)	0.0301 (8)	0.0004 (7)	0.0102 (6)	−0.0010 (6)
C10	0.0353 (9)	0.0274 (9)	0.0286 (7)	0.0023 (7)	0.0059 (6)	−0.0010 (6)
C5	0.0408 (10)	0.0297 (9)	0.0368 (9)	−0.0001 (7)	0.0099 (7)	−0.0050 (7)
C11	0.0493 (12)	0.0495 (11)	0.0348 (9)	0.0080 (9)	0.0030 (7)	−0.0025 (8)
C9	0.0557 (12)	0.0320 (10)	0.0350 (9)	−0.0030 (8)	0.0087 (7)	0.0008 (7)
N1	0.0506 (10)	0.0521 (10)	0.0490 (9)	0.0117 (8)	0.0078 (7)	0.0072 (7)
C6	0.0613 (13)	0.0550 (13)	0.0374 (9)	0.0009 (10)	0.0154 (8)	−0.0131 (9)
C13	0.0426 (11)	0.0482 (12)	0.0529 (10)	−0.0101 (9)	0.0139 (8)	−0.0064 (8)
C12	0.0451 (12)	0.0599 (14)	0.0512 (10)	−0.0036 (10)	0.0039 (8)	−0.0127 (9)
C1	0.0583 (14)	0.0653 (15)	0.0618 (12)	0.0154 (11)	−0.0001 (10)	0.0046 (10)
C7	0.0639 (13)	0.0400 (12)	0.0462 (10)	0.0107 (10)	0.0004 (9)	−0.0178 (9)
C4	0.0487 (12)	0.0639 (14)	0.0528 (11)	0.0017 (10)	0.0190 (9)	0.0024 (9)
C8	0.0639 (13)	0.0279 (10)	0.0518 (11)	−0.0024 (9)	−0.0024 (9)	−0.0018 (8)
C2	0.0448 (13)	0.0705 (16)	0.0731 (14)	0.0124 (11)	0.0012 (10)	−0.0182 (12)
C3	0.0452 (13)	0.0887 (18)	0.0779 (15)	0.0000 (12)	0.0269 (10)	−0.0126 (13)

### Geometric parameters (Å, º)

**Table:**

N3—C14	1.4635 (18)	N1—C1	1.336 (2)
N3—C11	1.467 (2)	C6—C7	1.367 (3)
N3—H3	0.878 (17)	C6—H6A	0.9300
N2—C10	1.3401 (18)	C13—C12	1.511 (2)
N2—C6	1.343 (2)	C13—H13A	0.9700
N4—C14	1.4638 (19)	C13—H13B	0.9700
N4—C13	1.468 (2)	C12—H12A	0.9700
N4—H4	0.921 (17)	C12—H12B	0.9700
C14—C5	1.527 (2)	C1—C2	1.361 (3)
C14—C10	1.546 (2)	C1—H1A	0.9300
C10—C9	1.379 (2)	C7—C8	1.372 (2)
C5—N1	1.334 (2)	C7—H7A	0.9300
C5—C4	1.381 (2)	C4—C3	1.384 (3)
C11—C12	1.514 (2)	C4—H4A	0.9300
C11—H11A	0.9700	C8—H8A	0.9300
C11—H11B	0.9700	C2—C3	1.365 (3)
C9—C8	1.373 (2)	C2—H2A	0.9300
C9—H9A	0.9300	C3—H3A	0.9300
			
C14—N3—C11	112.16 (12)	C7—C6—H6A	118.0
C14—N3—H3	107.7 (11)	N4—C13—C12	109.54 (14)
C11—N3—H3	108.3 (11)	N4—C13—H13A	109.8
C10—N2—C6	117.07 (15)	C12—C13—H13A	109.8
C14—N4—C13	113.39 (13)	N4—C13—H13B	109.8
C14—N4—H4	108.1 (11)	C12—C13—H13B	109.8
C13—N4—H4	110.8 (11)	H13A—C13—H13B	108.2
N3—C14—N4	110.86 (12)	C13—C12—C11	109.23 (14)
N3—C14—C5	109.51 (12)	C13—C12—H12A	109.8
N4—C14—C5	107.45 (13)	C11—C12—H12A	109.8
N3—C14—C10	107.83 (12)	C13—C12—H12B	109.8
N4—C14—C10	114.09 (12)	C11—C12—H12B	109.8
C5—C14—C10	106.97 (12)	H12A—C12—H12B	108.3
N2—C10—C9	121.72 (14)	N1—C1—C2	123.9 (2)
N2—C10—C14	117.23 (13)	N1—C1—H1A	118.0
C9—C10—C14	121.03 (13)	C2—C1—H1A	118.0
N1—C5—C4	122.15 (17)	C6—C7—C8	118.39 (16)
N1—C5—C14	115.30 (14)	C6—C7—H7A	120.8
C4—C5—C14	122.55 (15)	C8—C7—H7A	120.8
N3—C11—C12	113.15 (15)	C5—C4—C3	118.56 (19)
N3—C11—H11A	108.9	C5—C4—H4A	120.7
C12—C11—H11A	108.9	C3—C4—H4A	120.7
N3—C11—H11B	108.9	C7—C8—C9	118.52 (17)
C12—C11—H11B	108.9	C7—C8—H8A	120.7
H11A—C11—H11B	107.8	C9—C8—H8A	120.7
C8—C9—C10	120.09 (15)	C1—C2—C3	118.3 (2)
C8—C9—H9A	120.0	C1—C2—H2A	120.9
C10—C9—H9A	120.0	C3—C2—H2A	120.9
C5—N1—C1	117.65 (17)	C2—C3—C4	119.4 (2)
N2—C6—C7	124.09 (16)	C2—C3—H3A	120.3
N2—C6—H6A	118.0	C4—C3—H3A	120.3
			
C11—N3—C14—N4	−53.05 (17)	C10—C14—C5—C4	97.46 (17)
C11—N3—C14—C5	−171.44 (13)	C14—N3—C11—C12	53.15 (17)
C11—N3—C14—C10	72.50 (16)	N2—C10—C9—C8	3.5 (2)
C13—N4—C14—N3	56.96 (16)	C14—C10—C9—C8	−178.02 (15)
C13—N4—C14—C5	176.59 (11)	C4—C5—N1—C1	0.9 (3)
C13—N4—C14—C10	−65.00 (16)	C14—C5—N1—C1	−179.79 (15)
C6—N2—C10—C9	−2.7 (2)	C10—N2—C6—C7	−0.5 (3)
C6—N2—C10—C14	178.80 (14)	C14—N4—C13—C12	−58.22 (17)
N3—C14—C10—N2	−145.84 (13)	N4—C13—C12—C11	54.63 (19)
N4—C14—C10—N2	−22.22 (19)	N3—C11—C12—C13	−53.6 (2)
C5—C14—C10—N2	96.46 (16)	C5—N1—C1—C2	0.2 (3)
N3—C14—C10—C9	35.63 (19)	N2—C6—C7—C8	2.9 (3)
N4—C14—C10—C9	159.24 (14)	N1—C5—C4—C3	−1.2 (3)
C5—C14—C10—C9	−82.08 (17)	C14—C5—C4—C3	179.54 (16)
N3—C14—C5—N1	161.53 (13)	C6—C7—C8—C9	−2.0 (3)
N4—C14—C5—N1	41.03 (17)	C10—C9—C8—C7	−1.0 (2)
C10—C14—C5—N1	−81.87 (16)	N1—C1—C2—C3	−1.0 (3)
N3—C14—C5—C4	−19.1 (2)	C1—C2—C3—C4	0.6 (3)
N4—C14—C5—C4	−139.63 (16)	C5—C4—C3—C2	0.4 (3)

### Hydrogen-bond geometry (Å, º)

**Table:**

*D*—H···*A*	*D*—H	H···*A*	*D*···*A*	*D*—H···*A*
N3—H3···N2^i^	0.878 (17)	2.425 (18)	3.2845 (19)	166.4 (15)
N4—H4···N1	0.921 (17)	2.379 (17)	2.701 (2)	100.3 (12)
N4—H4···N2	0.921 (17)	2.564 (17)	2.8034 (19)	95.3 (12)

Symmetry code: (i) *x*, −*y*+1/2, *z*+1/2.
